# Supplementary material for: Spatial quantification of dynamic inter and intra particle crystallographic heterogeneities within lithium ion electrodes
Source: Nat Commun. 2020 Jan 31;11:631. doi: 10.1038/s41467-020-14467-x (PMC6994469; doi:10.1038/s41467-020-14467-x)
Supplement: Supplementary file 1 — Supplementary Information [file 41467_2020_14467_MOESM1_ESM.pdf]

Supplementary Information

Spatial quantification of dynamic inter and intra particle crystallographic heterogeneities within lithium ion electrodes

Finegan D.P. et al.

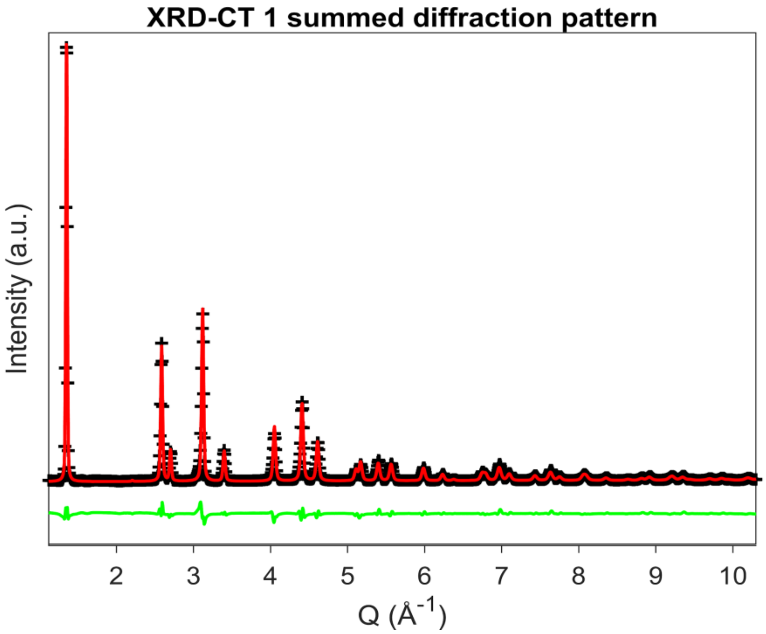

Supplementary Figure 1. Rietveld analysis of the summed diffraction pattern from the XRD-CT dataset 1. Black: Observed pattern, Red: calculated pattern, Green: difference plot.

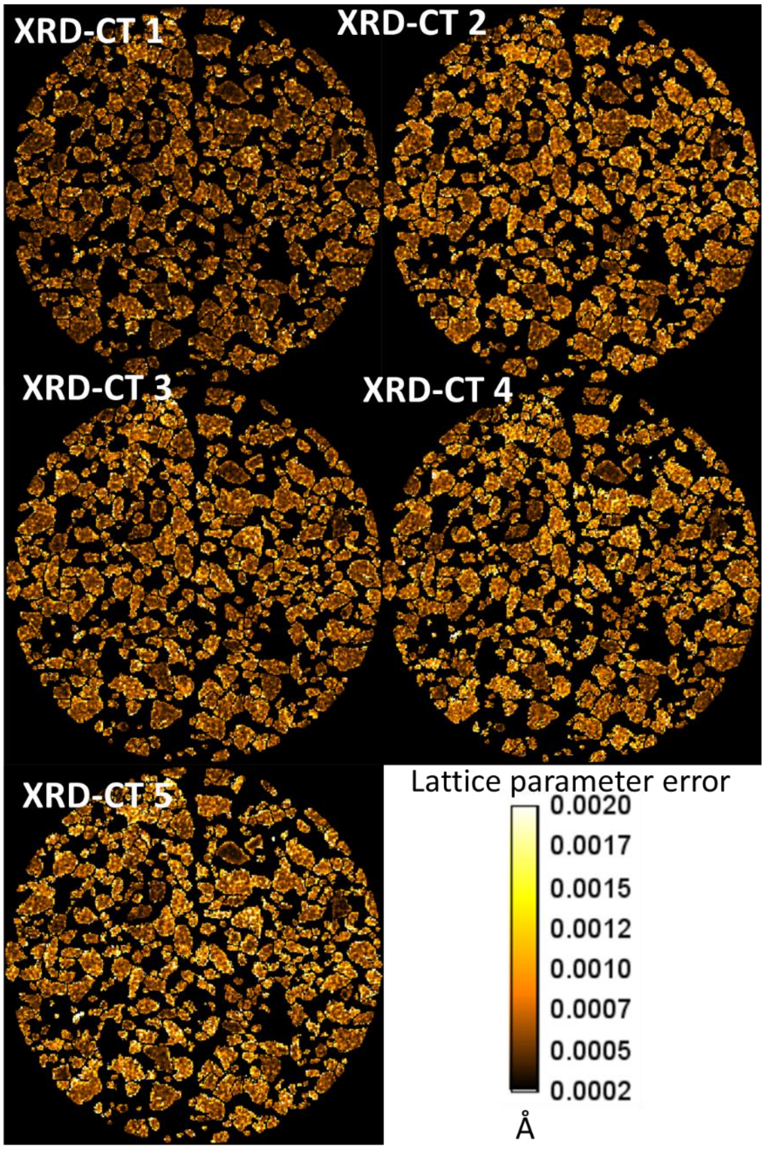

Supplementary Figure 2: Error values for the Rietveld refined lattice parameter maps shown for the operando cell in the main manuscript.

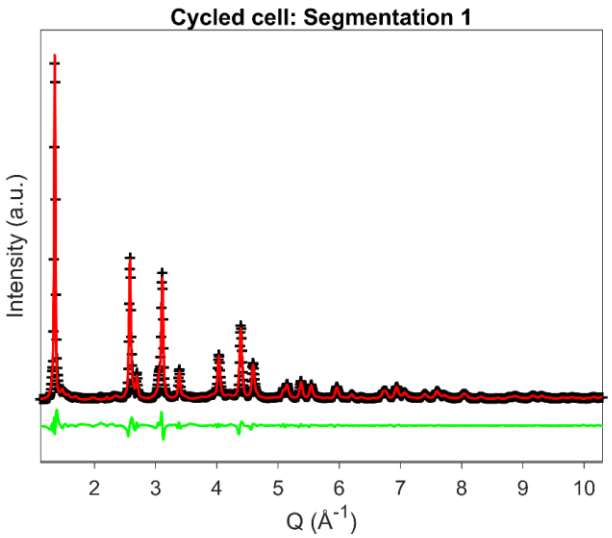

Supplementary Figure 3: Rietveld analysis of the summed diffraction pattern exported from Segmentation 1 of the XRD-CT data from the degraded cycled cell shown in Figure 7 of the main manuscript. Black: Observed pattern, Red: calculated pattern, Green: difference plot.

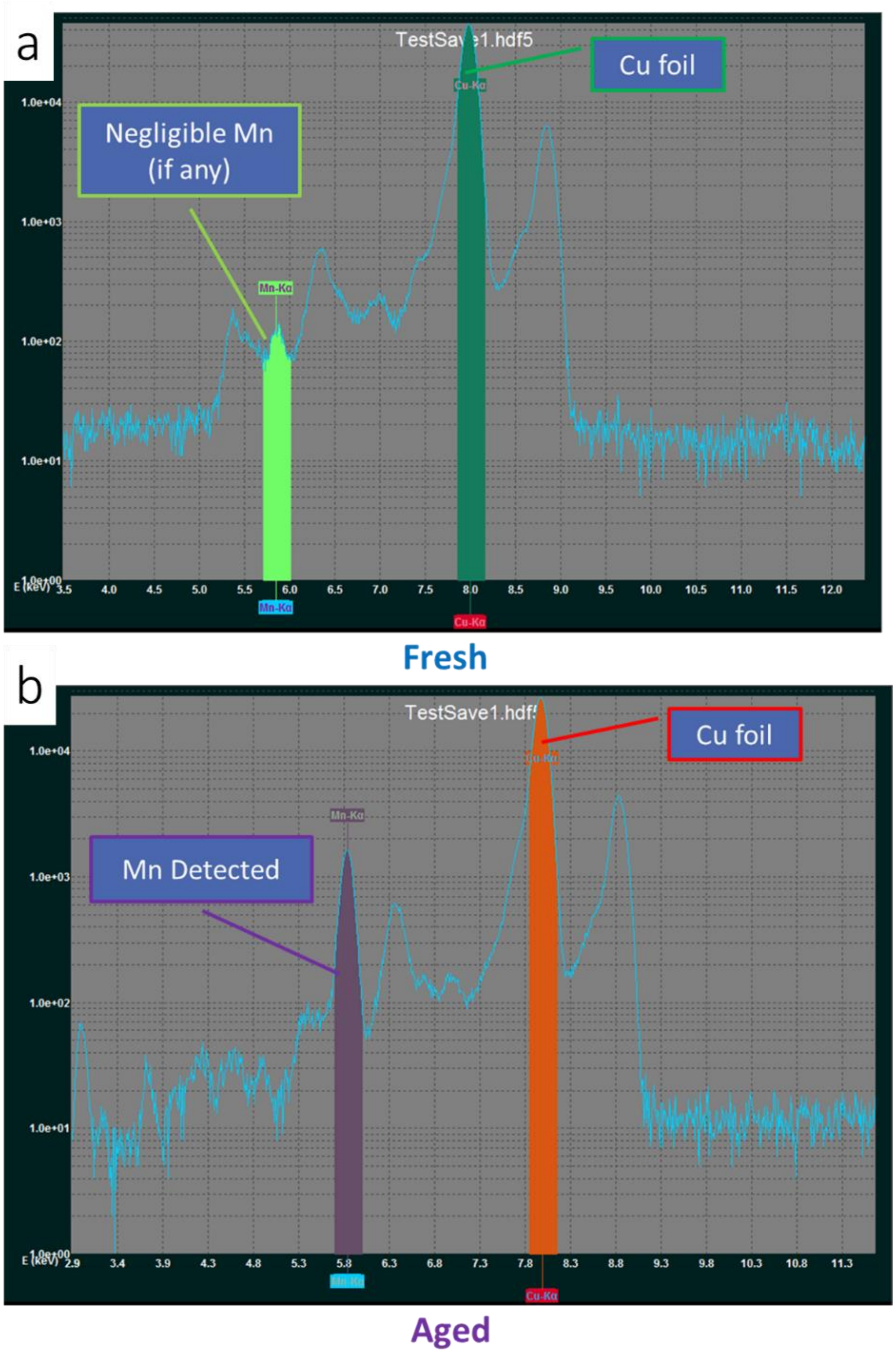

Supplementary Figure 4: X-ray fluorescence spectra of the graphite electrode from a fresh and degraded cycled LMO vs graphite.

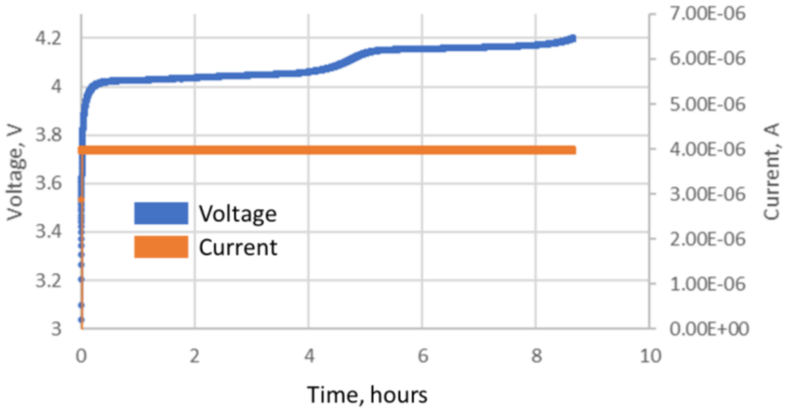

Supplementary Figure 5: Voltage and current profile of the operando cell during the initial charge step before the operando experiment.

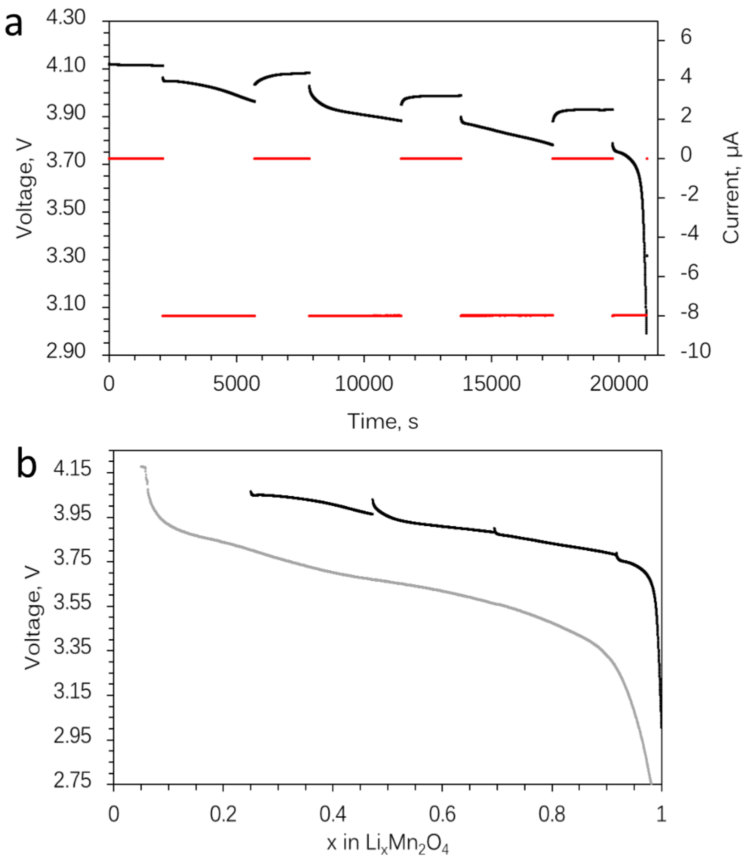

Supplementary Figure 6: (a) Voltage [black] and current [red] profile of the micro-cell during the XRD-CT experiment. The XRD-CT images were taken during the open circuit periods. (b) The voltage profile with respect to $Li_xMn_2O_4$ for the micro-cell with the open circuit periods removed [black] and a standard coin cell [gray]. The micro-cell contained Li as the counter electrode, whereas the coin cell contained graphite, which helps explain the discrepancy in voltage between the two.

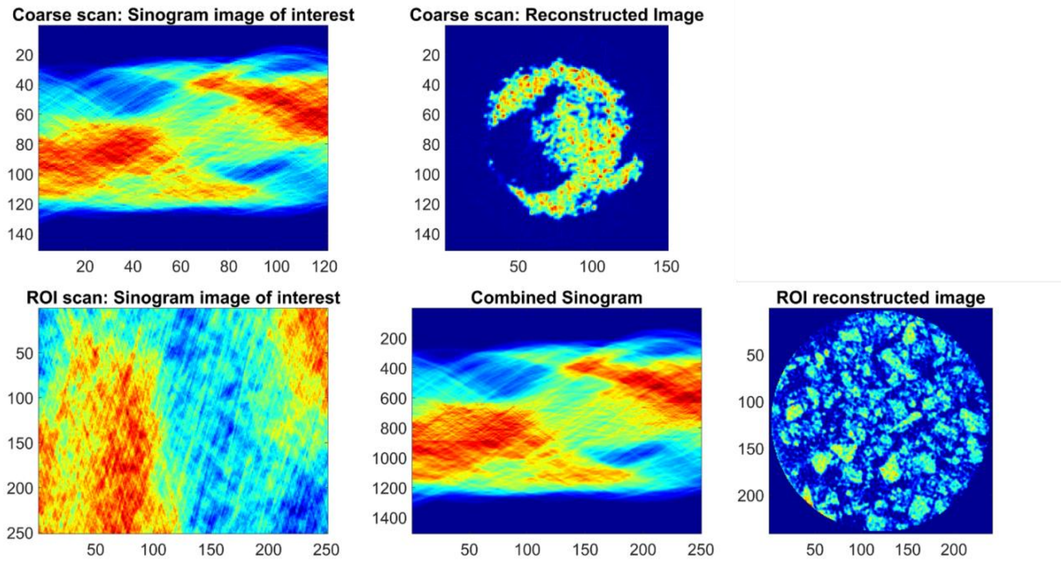

Supplementary Figure 7: Schematic representation of the data processing strategy followed to reconstruct the ROI XRD-CT data.

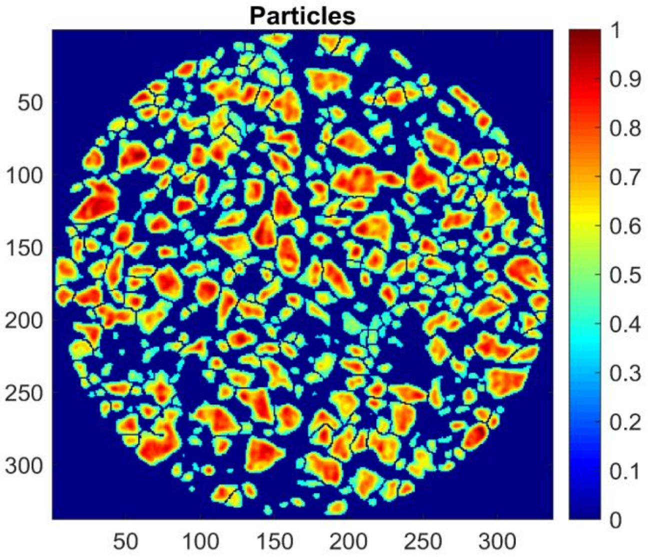

Supplementary Figure 8: Image derived from the manual segmentation of the sum of the $Li_xMn_2O_4$ phase distribution maps (i.e. five images in total).

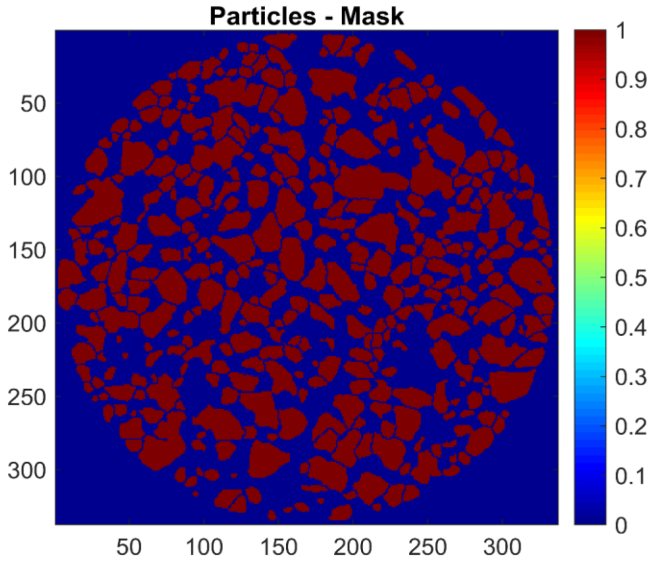

Supplementary Figure 9: Binary mask derived from the segmentation of the sum of the $Li_xMn_2O_4$ phase distribution maps (i.e. five images in total).

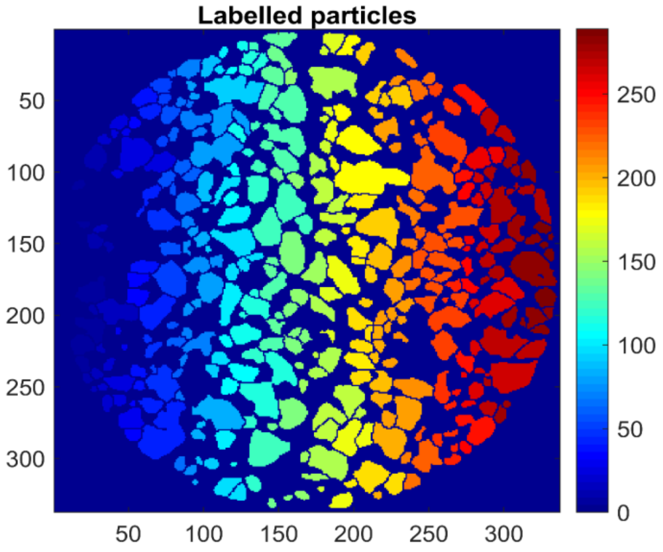

Supplementary Figure 10: Labelled particles calculated from the binary image.

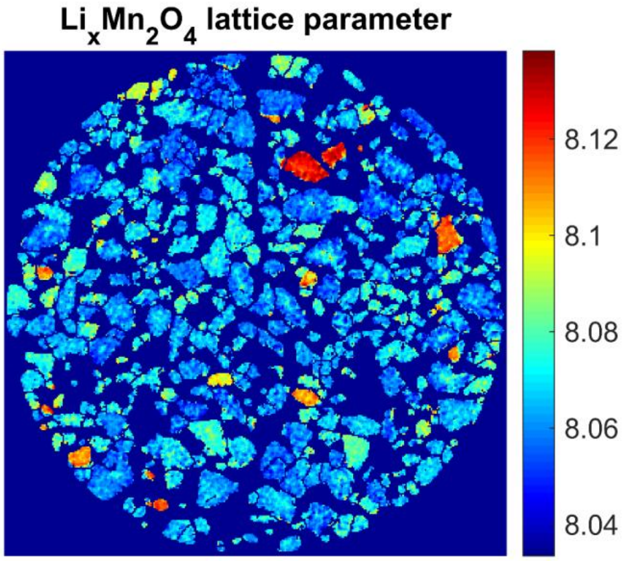

Supplementary Figure 11: LMO lattice parameter map obtained from the Rietveld analysis of XRD-CT dataset 1. Colorbar axis unit corresponds to Å.

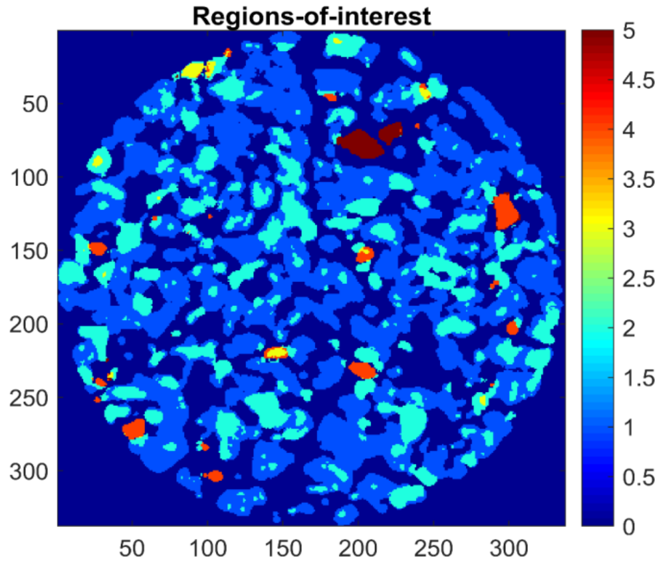

Supplementary Figure 12: Five segmented regions using the lattice parameter map. Colorbar axis corresponds to the label value, i.e. regions 1, 2, 3, 4 and 5.

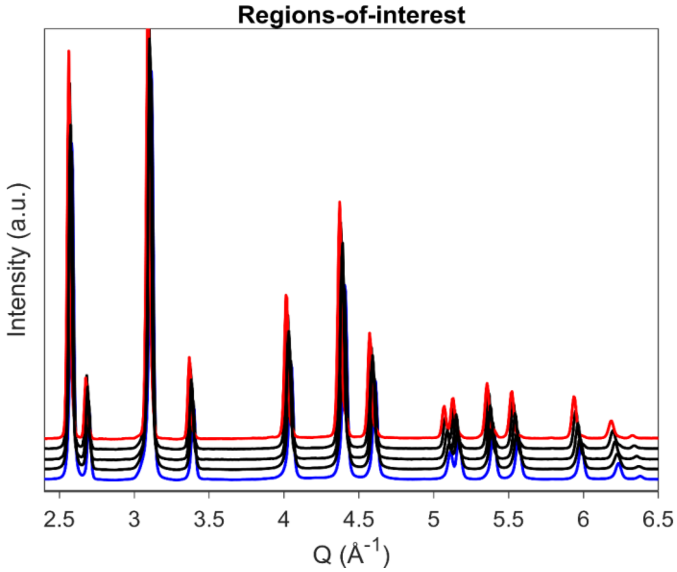

Supplementary Figure 13: Summed diffraction patterns from five regions-of-interest from XRD-CT dataset 1. Blue: Region 1, Red: Region 5.

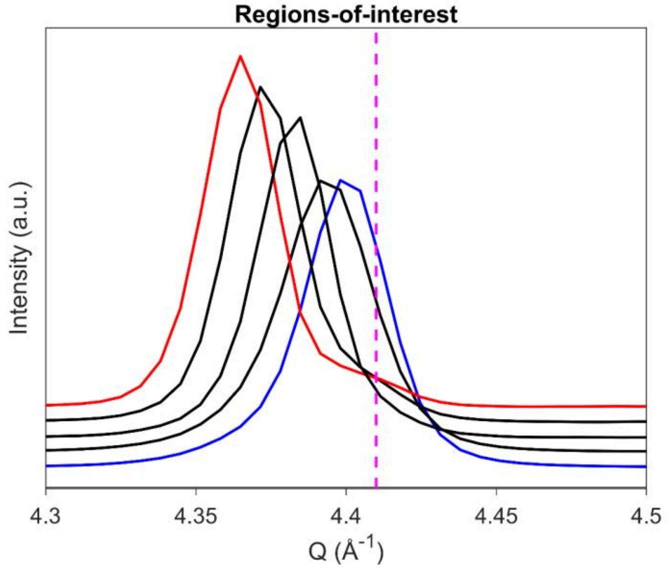

Supplementary Figure 14: Summed diffraction patterns from five regions-of-interest from XRD-CT dataset 1 (Blue: Region 1, Red: Region 5). There is a shoulder peak to all diffraction peaks corresponding to region 5.

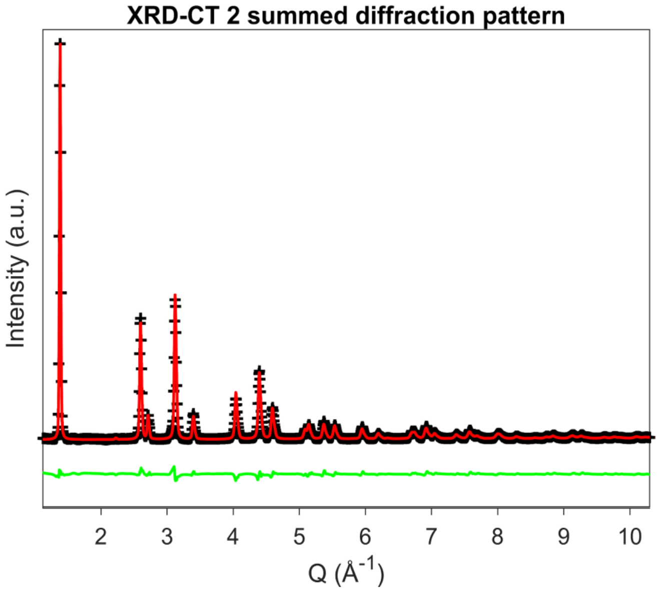

Supplementary Figure 15: Rietveld analysis of the summed diffraction pattern from the XRD-CT dataset 2 using both cubic $Li_xMn_2O_4$ and $Li_{0.5}Mn_2O_4$ phases. Black: Observed pattern, Red: calculated pattern, Green: difference plot.

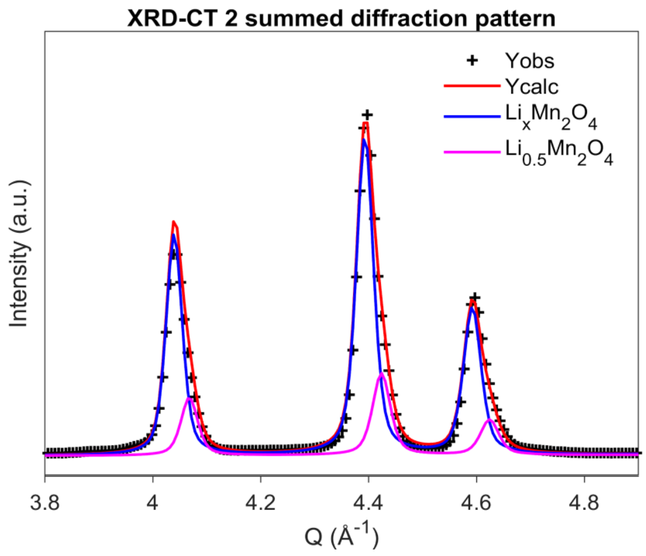

Supplementary Figure 16: Rietveld analysis of the summed diffraction pattern from the XRD-CT dataset 2 using both cubic $Li_xMn_2O_4$ and $Li_{0.5}Mn_2O_4$ phases. A region of interest is presented showing the individual contribution of these two phases.

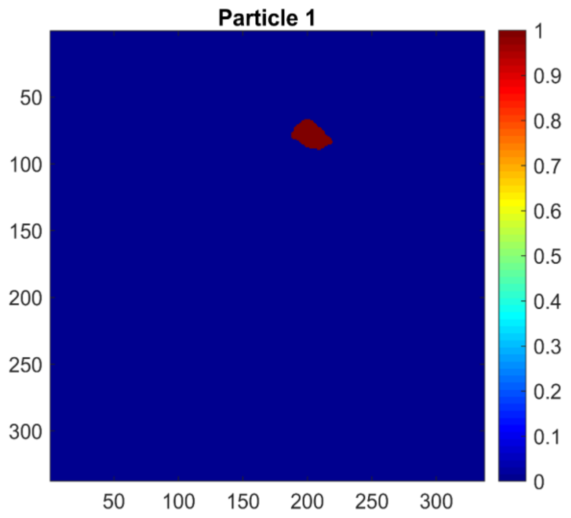

Supplementary Figure 17: Binary mask for Particle 1.

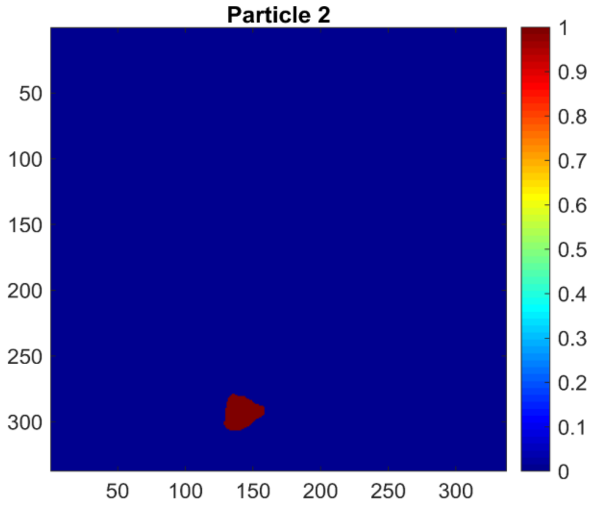

Supplementary Figure 18: Binary mask for Particle 2.

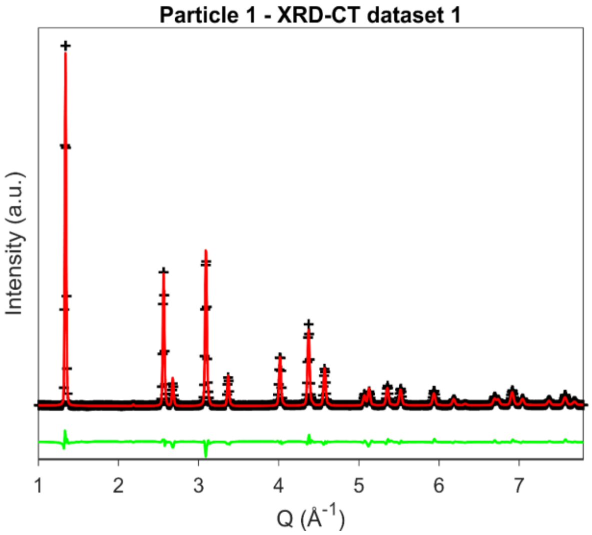

Supplementary Figure 19: Rietveld analysis of the summed diffraction pattern exported from the XRD-CT data after applying the binary mask for Particle 1. Black: Observed pattern, Red: calculated pattern, Green: difference plot.

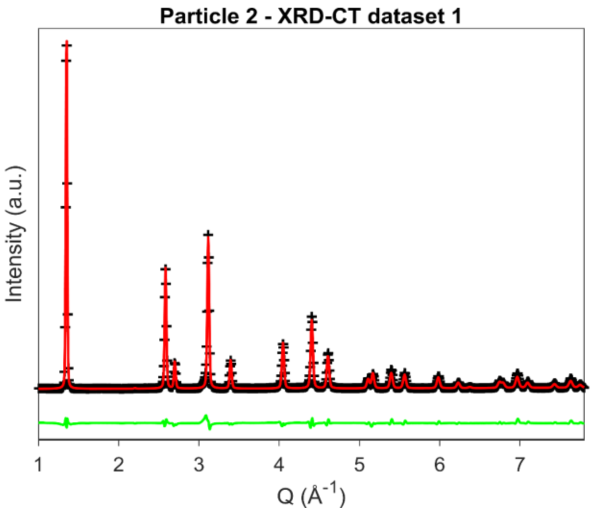

Supplementary Figure 20: Rietveld analysis of the summed diffraction pattern exported from the XRD-CT data after applying the binary mask for Particle 2. Black: Observed pattern, Red: calculated pattern, Green: difference plot.

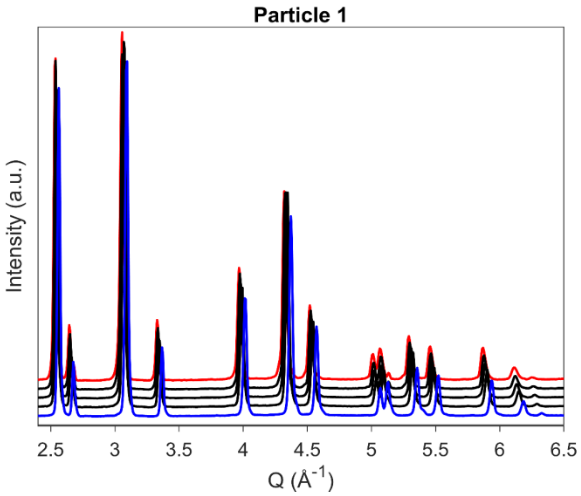

Supplementary Figure 21: Summed diffraction patterns from Particle 1 as derived from the five XRD-CT datasets (blue: dataset 1, red: dataset 5).

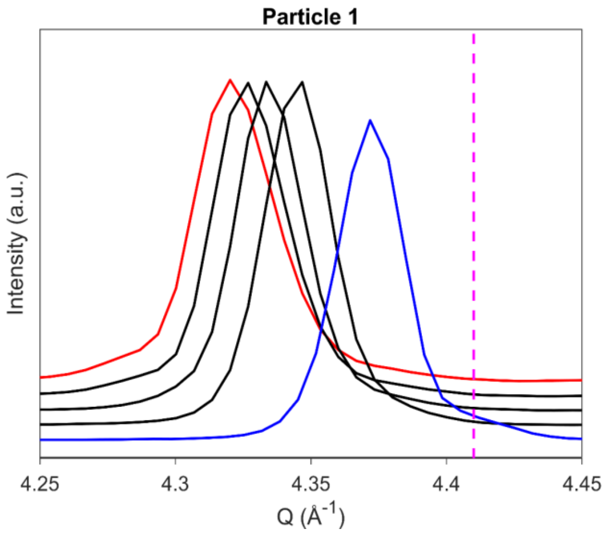

Supplementary Figure 22: A diffraction peak of interest from Particle 1 as derived from the five XRD-CT datasets (blue: dataset 1, red: dataset 5).

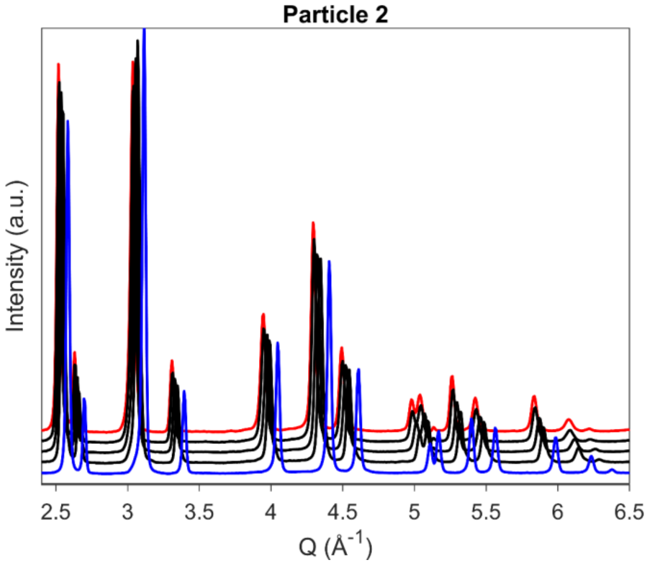

Supplementary Figure 23: Summed diffraction patterns from Particle 2 as derived from the five XRD-CT datasets (blue: dataset 1, red: dataset 5).

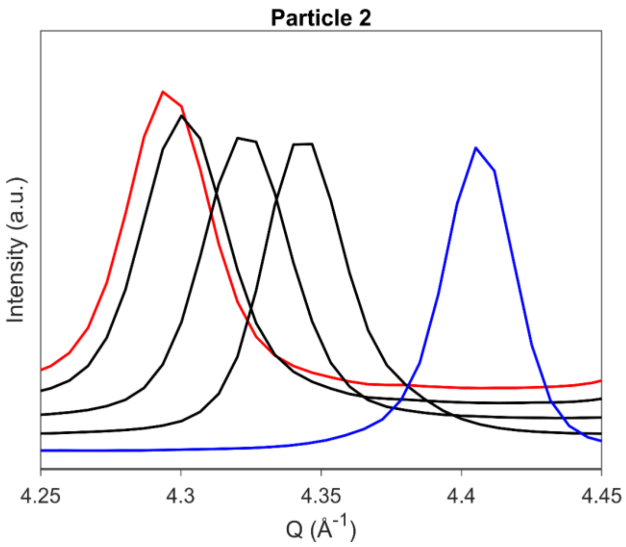

Supplementary Figure 24: A diffraction peak of interest from Particle 2 as derived from the five XRD-CT datasets (blue: dataset 1, red: dataset 5).

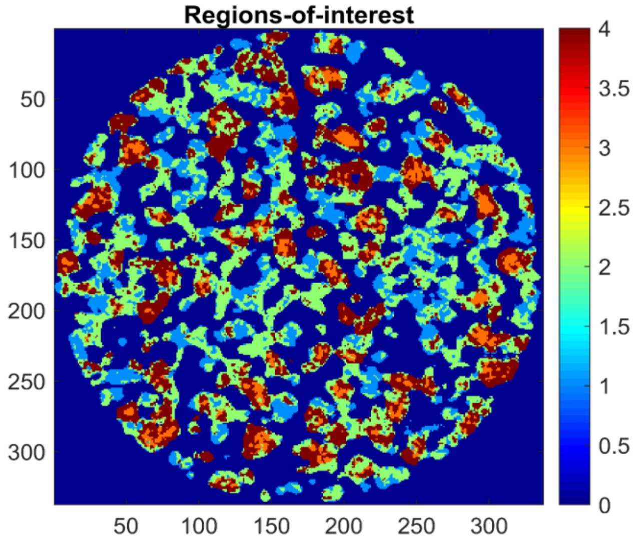

Supplementary Figure 25: Four segmented regions using the $LiMn_2O_4$ lattice parameter map as derived from the Rietveld analysis of XRD-CT dataset 2.

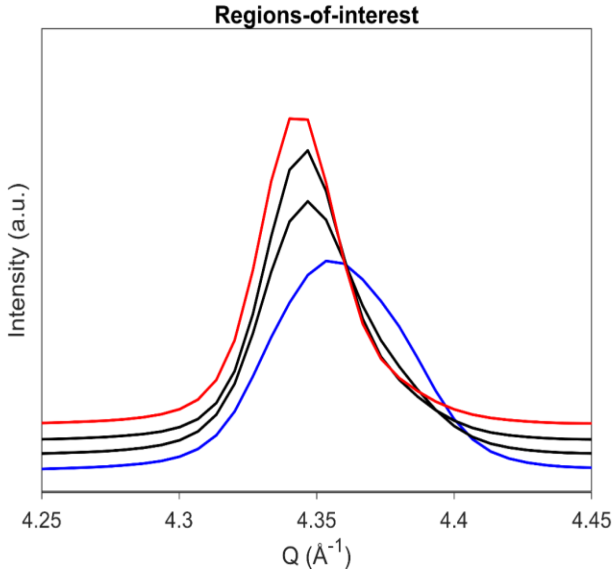

Supplementary Figure 26: Summed diffraction patterns from the four regions-of-interest from XRD-CT dataset 2. There is a shoulder peak to all diffraction peaks, especially to the ones corresponding to region 1 (blue line).

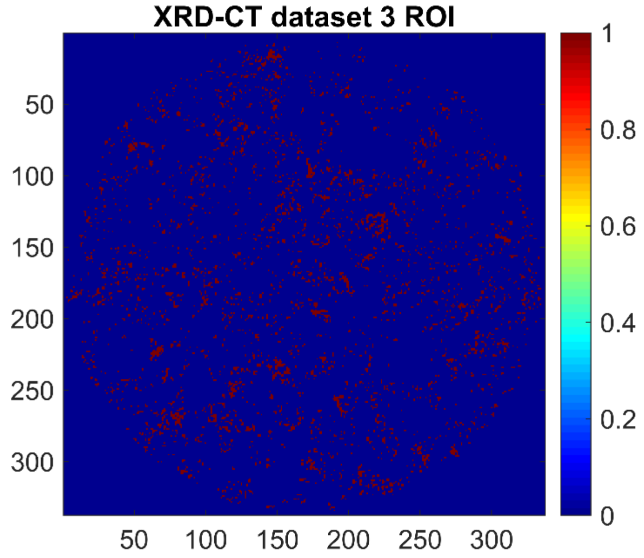

Supplementary Figure 27: Region of interest from XRD-CT dataset 3 which exhibits the abnormal behavior for the diffraction peak at *ca.* Q = 3.05 Å^{-1}.

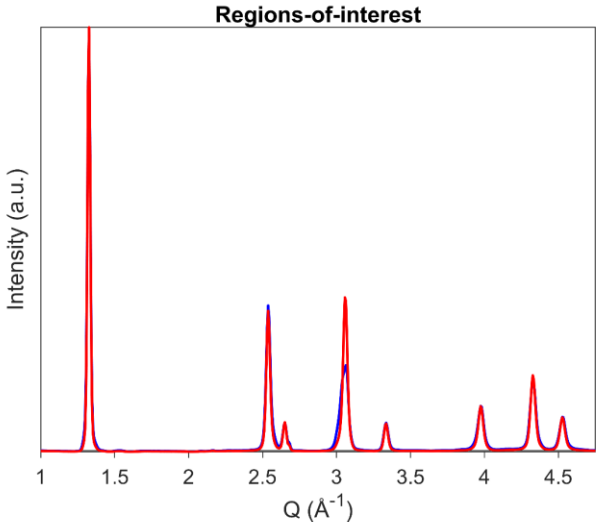

Supplementary Figure 28: Summed diffraction patterns from the two regions-of-interest from XRD-CT dataset 3. Blue: ROI presented in Supplementary Figure 26, Red: region corresponding to the inverse image presented in Supplementary Figure 26.

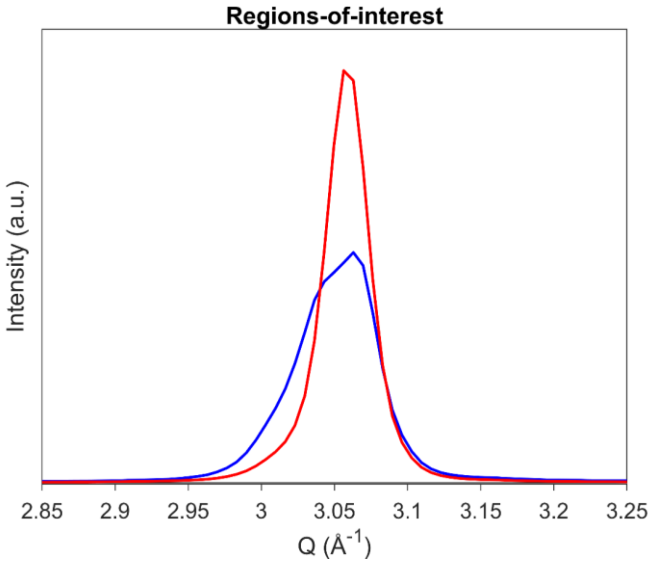

Supplementary Figure 29: Summed diffraction patterns from the two regions-of-interest from XRD-CT dataset 3 focusing on the (004) reflection. Blue: ROI presented in Supplementary Figure 26, Red: region corresponding to the inverse image presented in Supplementary Figure 26.

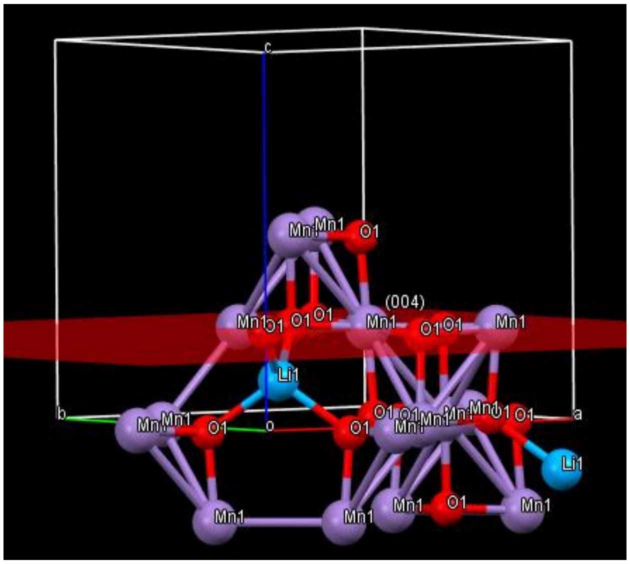

Supplementary Figure 30: Illustration of the cubic spinel $LiMn_2O_4$ structure using the Mercury software.

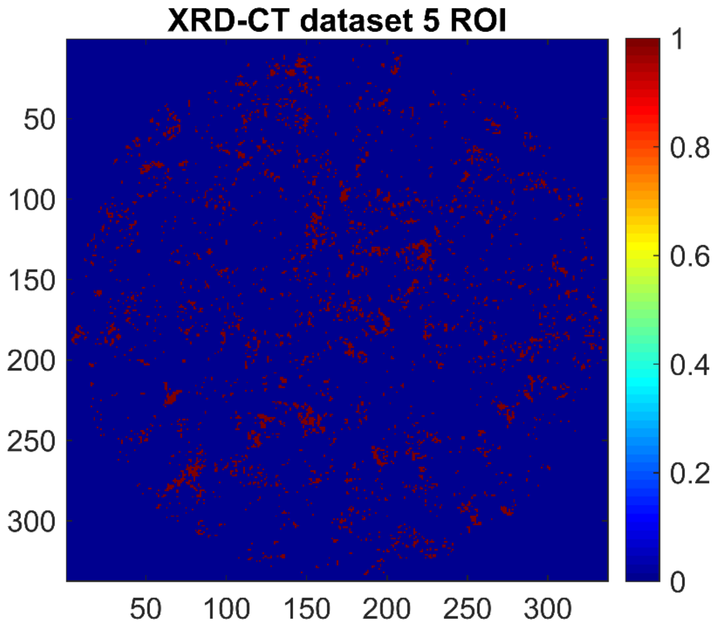

Supplementary Figure 31: Region of interest from XRD-CT dataset 5 which exhibits the abnormal behavior for the diffraction peak at *ca.* Q = 3.05 $Å^{-1}$.

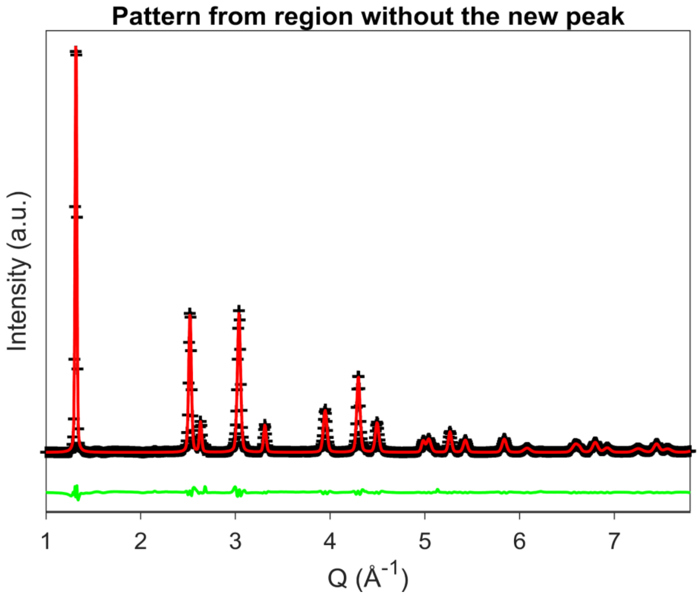

Supplementary Figure 32: Fit result using 23 pseudo-Voigt peaks for a diffraction pattern extracted from a region from XRD-CT dataset 5 which does not contain the new diffraction peak (region corresponding to the inverse image presented in Supplementary Figure 31). Black: Observed pattern, Red: calculated peaks, Green: difference plot.

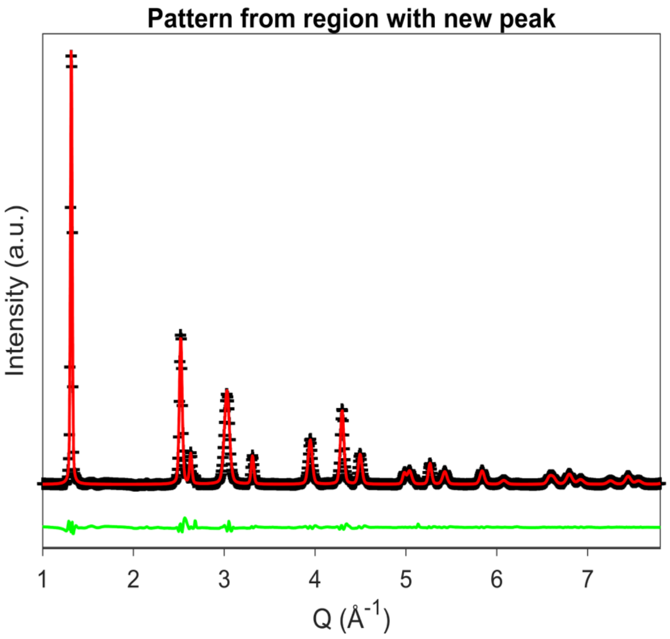

Supplementary Figure 33: Fit result using 23 pseudo-Voigt peaks for a diffraction pattern extracted from a region from XRD-CT dataset 5 which contains the new diffraction peak (ROI shown in Supplementary Figure 31).

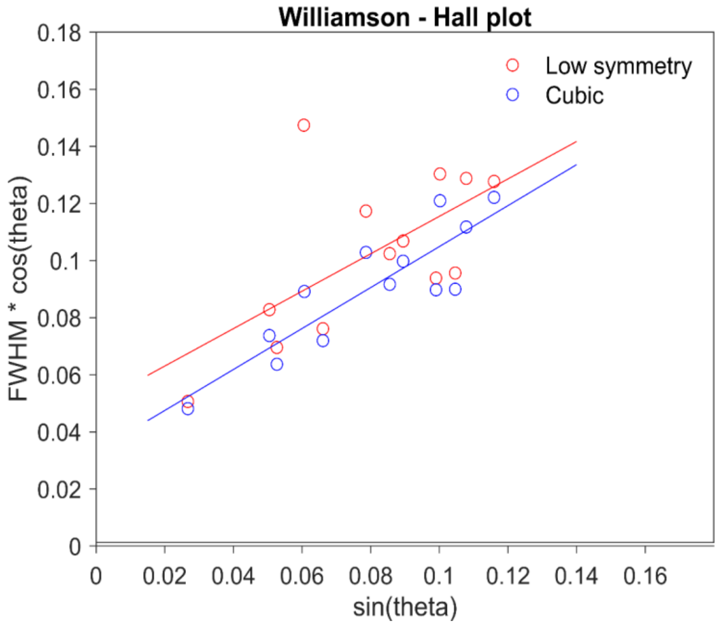

Supplementary Figure 34: Williamson-Hall plot using the peak shape parameter values from the 23 peaks obtained after fitting the two diffraction patterns of interest. Theta values in degrees (°).

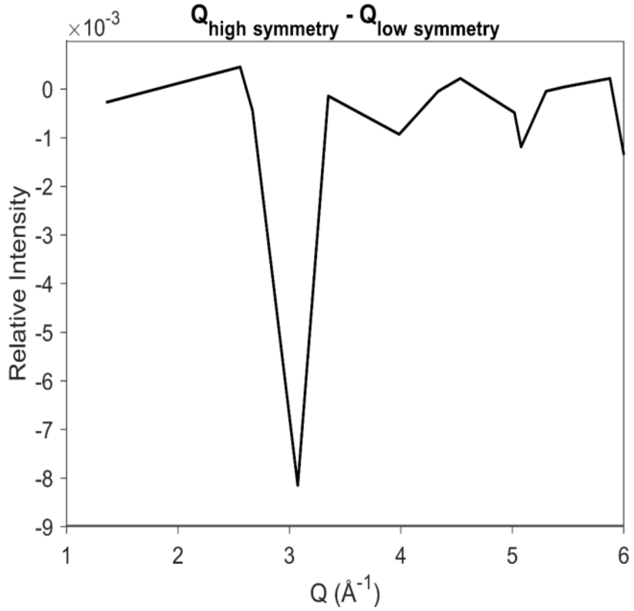

Supplementary Figure 35: The difference in Q position for certain peaks in the two diffraction patterns of interest.

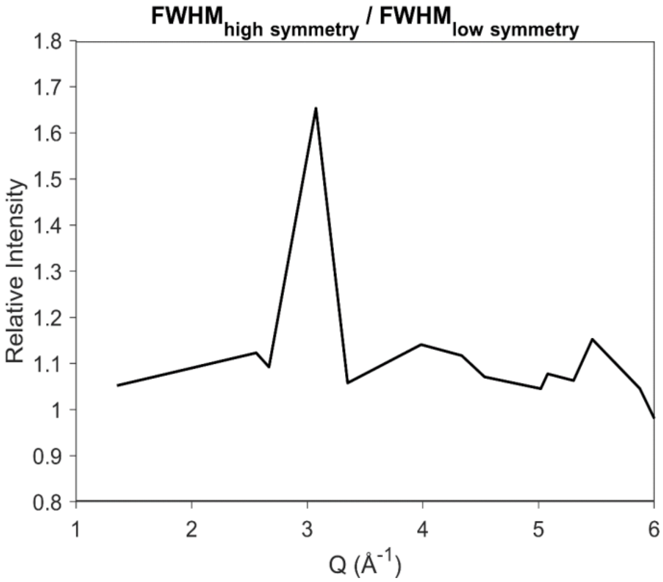

Supplementary Figure 36: The ratio of the FWHM of certain peaks in the two diffraction patterns of interest.

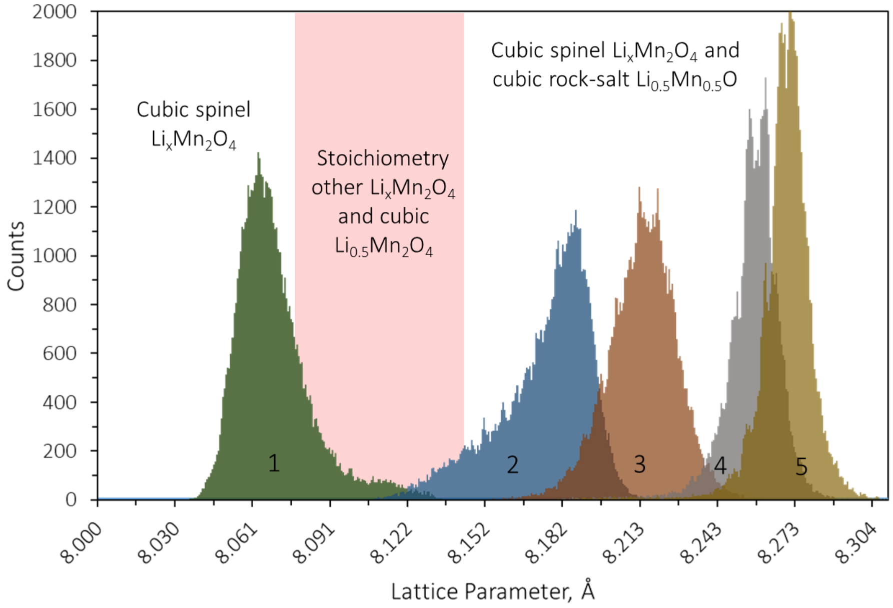

Supplementary Figure 37: Histograms of $LiMn_2O_4$ lattice parameter for the five XRD-CT datasets and the main phases present in these datasets.

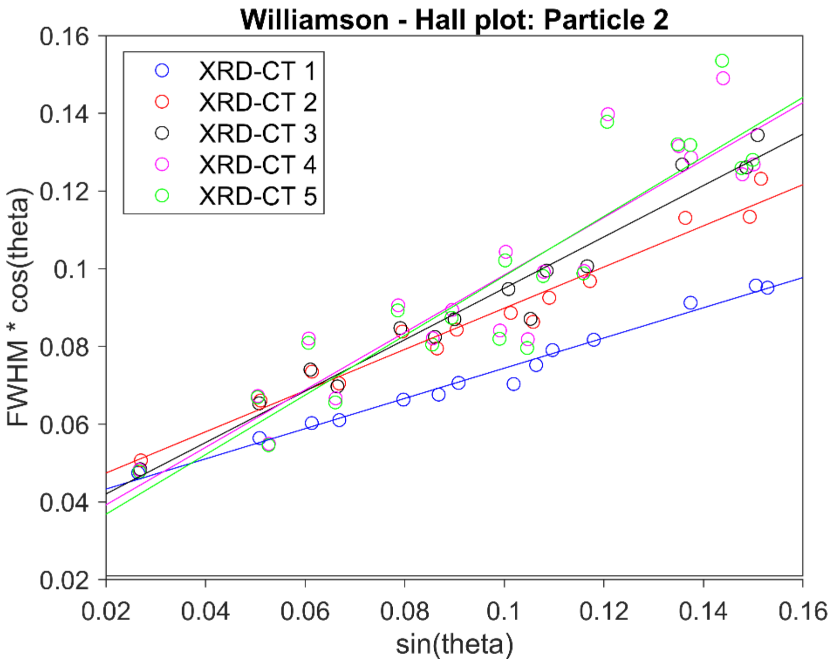

Supplementary Figure 38: Williamson-Hall plot using the peak shape parameter values from the peaks obtained after fitting the summed diffraction patterns from particle 2 for the five XRD-CT datasets. Theta values in degrees (°).

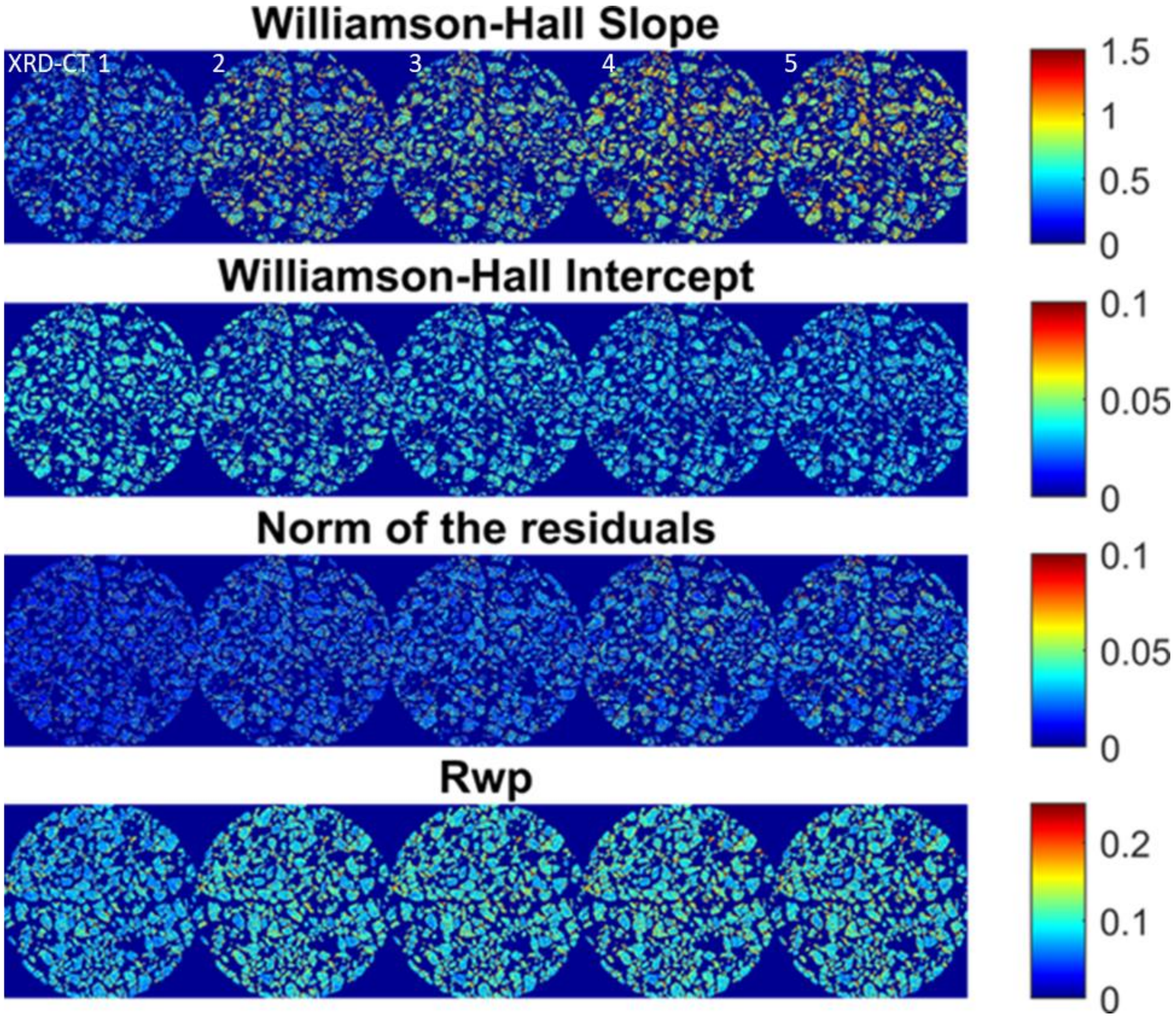

Supplementary Figure 39: Maps obtained from the Williamson-Hall plot spatially-resolved analysis using the five XRD-CT datasets. First row: Maps corresponding to the slopes of the fitted FWHM*cos(theta) vs sin(theta) lines. Second row: Maps corresponding to the intercepts of the fitted FWHM*cos(theta) vs sin(theta) lines. Third row: Maps corresponding to the norm of the residuals of the fitted FWHM*cos(theta) vs sin(theta) lines. Fourth row: Rwp maps corresponding to the error of the fitted Gaussian peaks during the spatially-resolved batch fitting.

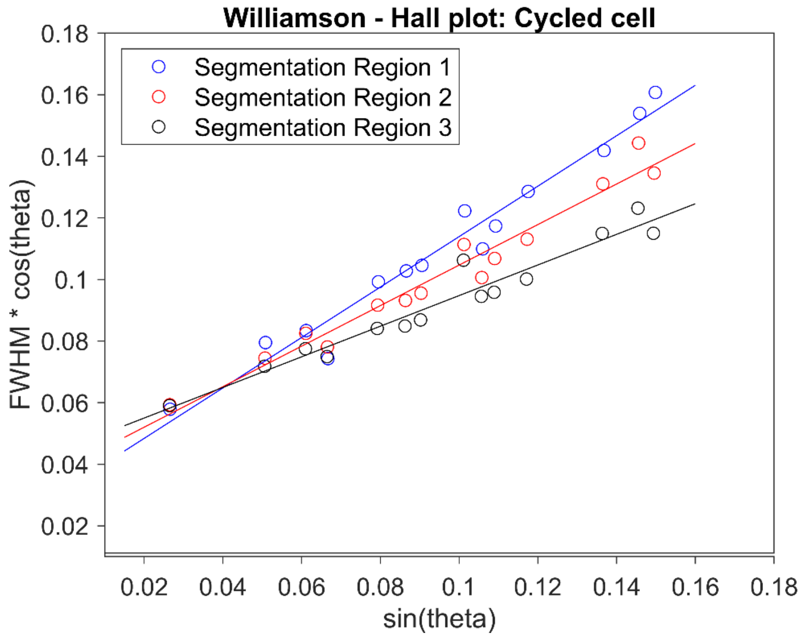

Supplementary Figure 40: Williamson-Hall plot using the peak shape parameter values from the peaks obtained after fitting the summed diffraction patterns from the three segmented regions of interest from the cycled cell in the main manuscript. Theta values in degrees (°).

Supplementary Table 1. Corresponding error values for quantification of mass fractions for Particle 1 in the main manuscript.

Depth 1		XRD-CT 1		XRD-CT 2		XRD-CT 3		XRD-CT 4		XRD-CT 5	
**Parameter**	**Phase**	**Value**	**Error**	**Value**	**Error**	**Value**	**Error**	**Value**	**Error**	**Value**	**Error**
**Mass fraction (%)**	Li0.5Mn2O4	6.178	0.489	6.280	0.77	3.135	1.207	1.94	0.964	2.14	1.29
**Mass fraction (%)**	LiMn2O4	90.091	0.592	90.580	0.76	90.337	1.24	91.73	1.039	88.98	1.242
**Mass fraction (%)**	LiMnO2	0	0	0.000	0.00	0.619	0.518	2.22	0.483	3.12	0.412
**Mass fraction (%)**	Li2MnO3	3.731	0.143	3.150	0.16	5.909	0.235	4.11	0.202	5.77	0.188
**Rwp**	-	5.917	-	5.655	-	8.205	-	7.61	-	6.693	-
**Lattice Parameter (Å)**	LiMn2O4	8.1305	0.0002	8.176	0.0003	8.1866	0.0006	8.194	0.0004	8.2009	0.0006
**Lattice Parameter (Å)**	Li0.5Mn2O4	8.0536	0.0023	8.119	0.00	8.1282	0.0103	8.1327	0.0134	8.1495	0.0154
**Lattice Parameter (Å)**	LiMnO2	-	-	-	-	4.1435	0.0091	4.15	0.0024	4.1536	0.0015

Depth 2		XRD-CT 1		XRD-CT 2		XRD-CT 3		XRD-CT 4		XRD-CT 5	
**Parameter**	**Phase**	**Value**	**Error**	**Value**	**Error**	**Value**	**Error**	**Value**	**Error**	**Value**	**Error**
**Mass fraction (%)**	Li0.5Mn2O4	2.54	0.247	2.39	0.42	4.610047	1.132	6.560716	1.17	6.40	1.366
**Mass fraction (%)**	LiMn2O4	97.46	0.247	95.79	0.43	94.94115	1.179	90.85574	1.19	91.57	1.39
**Mass fraction (%)**	LiMnO2	0	0	1.82	0.13	0.448801	0.373	2.583547	0.396	2.03	0.414
**Mass fraction (%)**	Li2MnO3	0	0	0.00	0.00	0	0	0	0.00	0.00	0
**Rwp**	-	4.294	-	4.81	-	5.521	-	5.71	-	6.424	-
**Lattice Parameter (Å)**	LiMn2O4	8.1309	0.0001	8.1806	0.0002	8.1986	0.0004	8.2104	0.0004	8.2175	0.0005
**Lattice Parameter (Å)**	Li0.5Mn2O4	8.0462	0.0032	8.1109	0.0055	8.1481	0.0064	8.1604	0.0044	8.1675	0.0054
**Lattice Parameter (Å)**	LiMnO2	-	-	-	-	4.1421	0.0096	4.1455	0.0017	4.1534	0.0023

Depth 3		XRD-CT 1		XRD-CT 2		XRD-CT 3		XRD-CT 4		XRD-CT 5	
**Parameter**	**Phase**	**Value**	**Error**	**Value**	**Error**	**Value**	**Error**	**Value**	**Error**	**Value**	**Error**
**Mass fraction (%)**	Li0.5Mn2O4	2.3	0.223	2.22	0.52	2.299	0.612	2.55	0.67	1.86	0.69
**Mass fraction (%)**	LiMn2O4	97.7	0.223	97.78	0.52	96.918	0.698	95.36	0.74	93.64	0.76
**Mass fraction (%)**	LiMnO2	0	0	0.00	0	0.783	0.351	2.09	0.317	4.50	0.38
**Mass fraction (%)**	Li2MnO3	0	0	0.00	0.00	0	0	0.00	0.00	0.00	0.00
**Rwp**	-	4.28	-	6.05	-	5.934	-	5.09	-	4.717	-
**Lattice Parameter (Å)**	LiMn2O4	8.1285	0.0001	8.1807	0.0003	8.2038	0.0003	8.221	0.0002	8.2331	0.0003
**Lattice Parameter (Å)**	Li0.5Mn2O4	8.0394	0.0032	8.1107	0.0074	8.1366	0.0081	8.16	0.0074	8.18	0.0105
**Lattice Parameter (Å)**	LiMnO2	-	-	-	-	4.1457	0.0056	4.16	0.0018	4.15	0.0015

Depth 4		XRD-CT 1		XRD-CT 2		XRD-CT 3		XRD-CT 4		XRD-CT 5	
**Parameter**	**Phase**	**Value**	**Error**	**Value**	**Error**	**Value**	**Error**	**Value**	**Error**	**Value**	**Error**
**Mass fraction (%)**	Li0.5Mn2O4	1.732	0.184	0.00	0.00	3.222	0.704	2.38	0.73	1.65	0.76
**Mass fraction (%)**	LiMn2O4	98.268	0.184	100.00	0.00	96.125	0.735	94.66	0.75	95.05	0.77
**Mass fraction (%)**	LiMnO2	0	0	0.00	0.00	0.653	0.233	2.96	0.25	3.30	0.24
**Mass fraction (%)**	Li2MnO3	0	0	0.00	0.00	0	0	0.00	0.00	0.00	0.00
**Rwp**	-	4.617	-	4.81	-	6.16	-	5.87	-	5.03	-
**Lattice Parameter (Å)**	LiMn2O4	8.1241	0.0001	8.18	0.00	8.2069	0.0003	8.2252	0.0003	8.24	0.0003
**Lattice Parameter (Å)**	Li0.5Mn2O4	8.0192	0.004	-	-	8.1443	0.0062	8.17	0.0086	8.1847	0.0127
**Lattice Parameter (Å)**	LiMnO2	-	-	-	-	4.1482	0.0074	4.15	0.0016	4.16	0.0013

Supplementary Table 2. Corresponding error values for quantification of mass fractions for Particle 2 in the main manuscript.

Depth 1		XRD-CT 1		XRD-CT 2		XRD-CT 3		XRD-CT 4		XRD-CT 5	
**Parameter**	**Phase**	**Value**	**Error**	**Value**	**Error**	**Value**	**Error**	**Value**	**Error**	**Value**	**Error**
**Mass fraction (%)**	LiMn2O4	100	0	94.012	0.47	96.579	0.371	95.99	0.508	94.72	0.48
**Mass fraction (%)**	Li0.5Mn2O4	-	-	5.988	0.46	-	-	-	-	-	-
**Mass fraction (%)**	LiMnO2	-	-	-	-	3.421	0.371	4.01	0.508	5.28	0.48
**Rwp**	Rwp	5.424	-	4.394	-	5.452	-	6.89	-	6.57	-
**Lattice Parameter (Å)**	LiMn2O4	8.0657	0.0002	8.187	0.0003	8.225	0.0003	8.27	0.0004	8.2779	0.0004
**Lattice Parameter (Å)**	Li0.5Mn2O4	-	-	8.117	0.00	-	-	-	-	-	-
**Lattice Parameter (Å)**	LiMnO2	-	-	-	-	4.161	0.0017	4.18	0.002	4.18	0.0014

Depth 2		XRD-CT 1		XRD-CT 2		XRD-CT 3		XRD-CT 4		XRD-CT 5	
**Parameter**	**Phase**	**Value**	**Error**	**Value**	**Error**	**Value**	**Error**	**Value**	**Error**	**Value**	**Error**
**Mass fraction (%)**	LiMn2O4	100	0	90.356	0.67	94.691	0.394	93.73	0.523	94.28	0.549
**Mass fraction (%)**	Li0.5Mn2O4	-	-	9.644	0.67	-	-	-	-	-	-
**Mass fraction (%)**	LiMnO2	-	-	-	-	5.309	0.394	6.27	0.523	5.72	0.549
**Rwp**	Rwp	4.827	-	4.165	-	4.393	-	6.15	-	6.70	-
**Lattice Parameter (Å)**	LiMn2O4	8.0659	0.0002	8.184	0.0003	8.2213	0.0002	8.27	0.0004	8.2777	0.0004
**Lattice Parameter (Å)**	Li0.5Mn2O4	-	-	8.124	0.00	-	-	-	-	-	-
**Lattice Parameter (Å)**	LiMnO2	-	-	-	-	4.1429	0.0013	4.17	0.0013	4.18	0.0014

Depth 3		XRD-CT 1		XRD-CT 2		XRD-CT 3		XRD-CT 4		XRD-CT 5	
**Parameter**	**Phase**	**Value**	**Error**	**Value**	**Error**	**Value**	**Error**	**Value**	**Error**	**Value**	**Error**
**Mass fraction (%)**	LiMn2O4	100	0	94.425	0.50	96.833	0.365	95.76	0.431	94.79	0.447
**Mass fraction (%)**	Li0.5Mn2O4	-	-	5.575	0.50	3.167	0.365	4.25	0.431	5.21	0.447
**Mass fraction (%)**	LiMnO2	-	-	-	-	-	-	-	-	-	-
**Rwp**	Rwp	4.631	-	5.005	-	5.402	-	6.68	-	6.02	-
**Lattice Parameter (Å)**	LiMn2O4	8.0702	0.0002	8.185	0.0003	8.223	0.0003	8.26	0.0003	8.2764	0.0003
**Lattice Parameter (Å)**	Li0.5Mn2O4	-	-	8.114	0.0027	-	-	-	-	-	-
**Lattice Parameter (Å)**	LiMnO2	-	-	-	-	4.1548	0.0024	4.17	0.0016	4.18	0.0016

Depth 4		XRD-CT 1		XRD-CT 2		XRD-CT 3		XRD-CT 4		XRD-CT 5	
**Parameter**	**Phase**	**Value**	**Error**	**Value**	**Error**	**Value**	**Error**	**Value**	**Error**	**Value**	**Error**
**Mass fraction (%)**	LiMn2O4	100	0	90.564	0.55	95.254	0.227	94.48	0.246	97.69	0.237
**Mass fraction (%)**	Li0.5Mn2O4	-	-	9.436	0.55	4.746	0.227	5.52	0.246	2.31	0.237
**Mass fraction (%)**	LiMnO2	-	-	-	-	-	-	-	-	-	-
**Rwp**	Rwp	5.538	-	4.427	-	4.611	-	6.026	-	7.135	-
**Lattice Parameter (Å)**	LiMn2O4	8.0718	0.0002	8.186	0.0003	8.2232	0.0002	8.2663	0.0003	8.278	0.0004
**Lattice Parameter (Å)**	Li0.5Mn2O4	-	-	8.117	0.0021	-	-	-	-	-	-
**Lattice Parameter (Å)**	LiMnO2	-	-	-	-	4.1516	0.0017	4.174	0.0013	4.1847	0.0021

Supplementary Table 3. Corresponding error values for quantification of mass fractions for the 3 segmentations of the degraded electrode sample in the main manuscript.

Segmentaion 1				
**Phase**	**Wt. %**	**Error**	**LPA**	**Error**
LiMn2O4	68.92	1.814	8.115	0.0006
LiMnO2	19.61	2.087	4.132	0.0016
Li2MnO3	11.47	0.49	-	-
Rwp (%)	11.243	-	-	-

Segmentation 2				
**Phase**	**Wt. %**	**Error**	**LPA**	**Error**
LiMn2O4	91.96	0.202	8.129	0.0004
LiMnO2	0	-	-	-
Li2MnO3	8.04	0.202	-	-
Rwp (%)	6.504	-	-	-

Segmentation 3				
**Phase**	**Wt. %**	**Error**	**LPA**	**Error**
LiMn2O4	94.39	0.245	8.140	0.0005
LiMnO2	0	-	-	-
Li2MnO3	5.61	0.245	-	-
Rwp (%)	7.719	-	-	-

Supplementary Note 1. Lattice parameter error

Operando cell

The quality of the fits obtained from the Rietveld analysis of the XRD-CT data was high. As an example, the fit from the Rietveld analysis of the summed diffraction pattern from XRD-CT dataset 1 is presented in Supplementary Figure 1 (Rwp = 5.731 %).

The error maps for the $Li_xMn_2O_4$ lattice parameter values, as calculated from the Topas v5 software, presented in Figure 3 and 4 in the main manuscript are shown in Supplementary Figure 2. The error in most regions is below 0.002 Å, indicating that the confidence in the lattice parameter values is very high.

For Rietveld quantification of the mass fractions present in the single particles (Particle 1 and Particle 2) and the degraded electrode shown in Figures 6 and 7 in the main manuscript, the corresponding error values are shown in Supplementary Tables 1 and 2. The process of Rietveld refinement is discussed in a later section.

Cycled cell

The quality of the fit from the Rietveld analysis of the summed diffraction pattern from Segmentation 1 in Figure 7 of the main manuscript was high (Rwp = 7.93 %). The result is shown in Supplementary Figure 3.

There were also some minor peaks that could not be assigned to any known phase after performing phase identification using the ICSD and ICCD PDF databases. Peak indexing was also attempted with the Topas software but did not yield any promising candidate unit cells. These minor peaks appear at the following Q positions: 1.5, 2.31, 2.36, 2.62, 3.32, 3.55, 3.75, 3.9 and 4.2 $Å^{-1}$.

A table listing the errors for the mass fractions (Wt. %) and lattice parameters in Å (LPA) of the different phases for the 3 segmentations of the degraded cycled sample shown in Figure 7 of the main manuscript is shown in Supplementary Table 3.

Supplementary Note 2. XRF spectra of the LMO graphite electrodes

The capacity fade of the degraded cell is presented in Figure 7 of the main manuscript. Using X-ray fluorescence (XRF), significant amounts of elemental Mn were found on the degraded graphite electrode, indicating dissolution, migration and deposition on the graphite, as seen in Supplementary Figure 4. The system used for XRF was an Attomap Micro (Sigray, CA, USA).

Supplementary Note 3. Voltage profiles from the operando cell with Li vs. LMO

The voltage profile of the operando cell during the initial charge step outside the beam is shown in Supplementary Figure 5. The voltage profile of the operando cell from during the experiment is shown in Supplementary Figure 6a where the XRD-CT data was gathered during the open-circuit periods. A comparison of this voltage profile (without the open-circuit periods) from the micro-cell with Li vs. LMO, and the voltage profile from a standard coin cell with graphite vs. LMO is presented in Supplementary Figure 6b. The voltage profiles are similar in shape, but the voltage of the operando cell is higher, which is attributed to the counter electrode being Li rather than graphite.

Supplementary Note 4. Reconstruction of region-of-interest (ROI) XRD-CT data

The data processing strategy used for the high-resolution region-of-interest (ROI) XRD-CT data was the same as in our previous work[1]. This sequence is shown in Supplementary Figure 7.

1. First, a coarse XRD-CT scan was performed. The translation step size for this coarse scan was 20 μm. This dataset can be reconstructed independently of the ROI scan to investigate the sample at a different length scale.
2. A ROI XRD-CT scan was acquired after the coarse scan. Here, the translation step size was 1 μm and the total area covered was 351 μm × 351 μm.
3. The sinogram data volume from the coarse and the ROI scans were combined to yield a new data volume. To achieve this and at the same time suppress the formation of any artefacts in the reconstructed data, the coarse XRD-CT sinogram data were expanded by a factor of 20 times (linear interpolation). This expansion factor is equal to the ratio of the translation step size used for the coarse scan over the translation step size used the ROI scan.
4. Each data point of each sinogram (i.e. coarse and high resolution) is associated to a beam monitor value (diode). The sinograms were normalized with respect to the recorded beam intensity values leading to two sinograms with matching intensities. In case the beam intensity cannot be monitored, a scale factor has to be applied to the expanded coarse sinogram data so that the intensity between the two datasets is matched (i.e. to take into account the intensity mismatch between the two datasets).
5. The new combined sinogram data volume is reconstructed and to minimize the required time, only a region of diameter equal to the size of the ROI scan is reconstructed (351 pixels in this experiment).

Supplementary Note 5. Segmentation of the LMO particles in the XRD-CT data

The signal-to-noise ratio was not high enough to perform automatic segmentation to separate the particles/agglomerates from the background. In order to overcome this problem, a global $Li_xMn_2O_4$ image was calculated from the sum of the $Li_xMn_2O_4$ phase distribution maps (i.e. five images in total) as derived from the Rietveld analysis of the respective XRD-CT datasets. This image was then filtered using a mean filter and then a manual segmentation was performed. The result of the segmentation is shown in Supplementary Figure 8. A binary mask was created based on the results of the segmentation process. The mask is shown in Supplementary Figure 9. The particles were then labelled showing that a total number of ca. 290 particles are present in the XRD-CT ROI images. The results are presented in Supplementary Figure 10.

Supplementary Note 6. Identification of the secondary cubic LMO phase

This mask was then applied to the $Li_xMn_2O_4$ lattice parameter maps obtained from the Rietveld analysis of the in situ LMO XRD-CT data which led to clearer images. This is shown in Supplementary Figure 11 where one can clearly observe that different particles exhibit different values for $Li_xMn_2O_4$ lattice

parameter. This image was then imported into the Ilastik software[2] and image segmentation was performed using supervised machine learning. In total five regions-of-interest (ROIs) displaying different lattice parameter values were selected. The results are shown in Supplementary Figure 12. The summed diffraction patterns from these five regions are presented in Supplementary Figure 13. The same diffraction peaks are present in the summed diffraction patterns exported from all five regions but there is a shift to lower scattering angles (i.e. increase in unit cell size). This increase in unit cell size corresponds to different amounts of Li being incorporated in the unit cell. However, as shown in Supplementary Figure 14 there is shoulder peak to the right of all diffraction peaks corresponding to region 5. The following structures (ICSD codes in brackets) were used to model the diffraction pattern from region 5 but none of them yielded satisfactory results:

$Li_2Mn_2O_4$ (201557), Li_2MnO_2 (37327), Li_2MnO_3 (239796), $LiMn_2O_4$ (50415 – cubic spinel and 87774 – tetragonal), $LiMn_3O_4$ (201556), $LiMnO_2$ (81053 – orthorhombic and 173138 - rhombohedral), Li_2O (54368), Li_2O_2 (24143), Mn_2O_3 (290640 – orthorhombic, 236255 – triclinic, 236254 – rhombohedral, 9091 – cubic, 9090 – orthorhombic), Mn_2O_7 (60821 – monoclinic), Mn_3O_4 (68174 – tetragonal, 40110 – orthorhombic, 30005 – orthorhombic), Mn_5O_8 (16956 – monoclinic), MnO (657304 and 9864 – cubic, 262928 – hexagonal), MnO_2 (78331 – orthorhombic, 20229 – tetragonal, 248069 – orthorhombic, 78331 – orthorhombic, 393 – tetragonal, 54114 – orthorhombic and 76430 – hexagonal).

It was realized that the shoulder peaks correspond to a separate phase rather than a lower symmetry LMO phase than the cubic spinel $Li_xMn_2O_4$. As the shoulder peaks appear on the right-hand side of all $Li_xMn_2O_4$ diffraction peaks, this was new phase was assigned to another cubic $Li_xMn_2O_4$ phase. It should be noted that peak indexing of this minor phase was also attempted using Topas software but it was not possible to yield any useful results (peak indexing after assuming that all the peaks belong to the same phase did not give any promising candidate unit cells). Our observation is in agreement with the work of Bianchini et al.[3, 4] who reported the presence of metastable cubic $Li_{0.5}Mn_2O_4$ ($P2_13$ space group). The addition of the cubic $Li_{0.5}Mn_2O_4$ unit cell to the Rietveld model led to superior fits. The quality of the fit of the summed diffraction pattern from XRD-CT dataset 2 is shown in Supplementary Figure 15 (Rwp = 4.563 %). In Supplementary Figure 16 it is clearly demonstrated why the addition of the $Li_{0.5}Mn_2O_4$ phase is necessary and that it helps model the right-hand shoulder asymmetry very well.

Supplementary Note 7. Behavior of two distinct particles during lithiation

To investigate the heterogeneity in chemistry between distinct particles in the LMO electrode, two masks were created (Supplementary Figures 17 and 18), focusing on the two particles of interest discussed in the manuscript. The diffraction pattern from Particle 1, which contains the extra peaks, can be modelled well by using two cubic $Li_xMn_2O_4$ unit cells to model the data. Phase 1 shows lattice parameter equal to 8.13 Å while phase 2 shows lattice parameter equal to 8.047 Å. The results are shown in Supplementary Figure 19 (Rwp = 5.26%). The quality of the fit from the Rietveld analysis of the particle 2 is also high (Rwp = 4.62 %). The result is shown in Supplementary Figure 20. The summed diffraction patterns from Particle 1 from the five XRD-CT datasets are shown in Supplementary Figure 21. No new peaks appear during the lithiation experiment. Interestingly though, the shoulder peaks seem to disappear after XRD-CT dataset 1. It should be noted that inspection of the raw 2D diffraction data did not shown any indication of apparent strain. The presence of the shoulder peak can be more easily seen in Supplementary Figure 22 where a narrow Q range is used.

The summed diffraction patterns from Particle 2 from the five XRD-CT datasets are shown in Supplementary Figure 23. As shown in Supplementary Figure 24, Particle 2 behaves "normally" for the duration of the experiment. No new peaks or shoulder peaks appear and the peaks shift towards lower scattering angles showing that the particle is continuously being lithiated and the corresponding unit cell expanding during the experiment. A similar data processing strategy was used for XRD-CT dataset 2. Specifically, first the $Li_xMn_2O_4$ lattice parameter map was used to segment different regions-of-interest as shown in Supplementary Figure 25. The summed diffraction patterns from these four regions are shown in Supplementary Figure 26. Region 1 (blue peak) corresponds to the particles exhibiting the lowest lattice parameter value (between 8.1 – 8.15 Å). There is a right-hand shoulder present in these data which is in agreement with the observations from Particle 1 from XRD-CT dataset 1.

Supplementary Note 8. Identification of the cubic rock-salt $LiMnO_2$ phase

XRD-CT datasets 3, 4 and 5 did not reveal the presence of any other peaks/phases. However, careful inspection of the data showed that there are regions in the sample where the intensity of the $Li_xMn_2O_4$ diffraction peak at *ca.* Q = 3.05 $Å^{-1}$ shows abnormal behavior. The region of interest from XRD-CT dataset 3 which exhibits the abnormal behavior for the diffraction peak is shown in Supplementary Figure 27.

It is important to note that this peak corresponds to the (004) reflection. As shown in Supplementary Figures 28 and 29, the intensity of the rest of the diffraction peaks remain the same. It is also clearly shown that there is more than one peak present in this region. This implies that the cubic symmetry is broken and indeed it is not possible to obtain a good fit using a single cubic spinel $LiMn_2O_4$ unit cell.

Interestingly, as shown in Supplementary Figure 20 (created with the Mercury software), there are no Li atoms present in the unit cell at the (004) plane. However, one can imagine that the place of interstitial atoms and the expansion along the z axis can lead to a unit cell with lower symmetry. It has previously been suggested that the cubic spinel-type $Li_xMn_2O_4$ changes to first to a cubic rock-salt and then to a tetragonal phase during lithiation[5, 6].

To investigate this further, the two summed diffraction patterns from two regions of interest from XRD-CT dataset 5 were investigated in more detail. The region of interest from XRD-CT dataset 5 which exhibits the abnormal behavior for the diffraction peak is shown in Supplementary Figure 31. The properties of the peaks in the two summed diffraction patterns were examined using the Fityk software[7]. A pseudo-Voigt peak shape function was used to model the peaks present in two diffraction patterns exported from two different regions from XRD-CT dataset 5. Specifically the following 23 peak positions were used (in Q (Å-1)) :

1.35, 2.56, 2.67, 3.07, 3.35, 3.98, 4.34, 4.53, 5.02, 5.08, 5.30, 5.46, 5.88, 6.12, 6.63, 6.67, 6.84, 6.96, 7.29, 7.48, 7.60, 7.92 and 8.18

As expected the fits are excellent (i.e. since there is no chemistry to constraint the relative peak intensities) and the results are presented in Supplementary Figures 32 and 33. A Williamson-Hall plot was constructed using the results of the fits (i.e. position of the peaks and their full width at half maximum (FWHM))obtained from the Fityk software (Supplementary Figure 34). In Supplementary Figure 35, the shift of the peaks is presented. Specifically, the difference in Q position for each peak in the two diffraction patterns is calculated. In general, the values are negative implying that the peaks from the lower symmetry peak are shifted towards lower scattering angles. As expected, the difference is most significant

at the (004) reflection. Similarly, the ratio of the FWHM of each peak was also calculated. It can be clearly seen that the values for the lower symmetry phase are more than 1, indicating that all peaks have become broader. As expected, the peak corresponding to the (004) reflection is significantly broader.

The analysis led us to the conclusion that the observed left-hand side shoulder of the peak corresponding to the (004) reflection of the cubic spinel $Li_xMn_2O_4$ is attributed to the presence of the cubic rock-salt $LiMnO_2$ (ICSD: 194998) phase ($Li_{0.5}Mn_{0.5}O$). This phase generates its main diffraction peak at this Q region. The addition of this structure to the Rietveld model led to an improved fit and it was possible to account for the left-hand side peak asymmetry at ca. Q = 3.05 $Å^{-1}$.

Supplementary Note 9. Strain analysis for the $LiMn_2O_4$ phase

The same analysis was performed for the summed diffraction patterns from particle 2 from the five XRD-CT datasets. Particle 2 was chosen for the strain analysis as it is representative of the average electrode particles (in contrast to particle 1). As previously, the peak fitting was performed using the Fityk software using pseudo-Voigt functions to fit the $LiMn_2O_4$ peaks and considering the instrumental broadening of the peaks using the peak profile calculated using the CeO_2 pattern. The results are presented in Supplementary Figure 38 and it can be clearly seen that the slope of the fitted lines increases from XRD-CT dataset 1 to 5 while the offset remains stable. This result clearly demonstrates that in this case it is possible to decouple the crystallite size-strain effects. The broadening of the $LiMn_2O_4$ peaks is attributed to the increase of strain which could be caused by a variety of reasons including phase segregation (the formation of the $LiMnO_2$ rock-salt phase is observed from XRD-CT dataset 2) and the lithiation process itself. The values for the slopes for the five datasets were 0.39, 0.53, 0.66, 0.74 and 0.77 respectively. It should be noted that, as shown in Supplementary Figure 38, the fit is worse for XRD-CT datasets 4 and 5 as the points deviate more from the linear model implying the presence of other features in the $LiMn_2O_4$ peaks (this coincident with Li occupancy exceeding 1).

It was also attempted to perform this analysis in a spatially-resolved manner. As the signal-to-noise ratio is worse for the spatially-resolved diffraction patterns present in the reconstructed XRD-CT data, only the first seven $LiMn_2O_4$ diffraction peaks (corresponding to hkl reflections: (111), (311), (222), (004), (331), (511), (044) and 442) were used for the analysis. We developed in-house python code for batch multi-peak fitting using the scipy.optimise package using gaussian peak shapes (http://pd.chem.ucl.ac.uk/pdnn/peaks/gauss.htm). Initially, the peak fitting was performed using the CeO_2 pattern and the FWHM of the peaks was calculated. The 2theta broadening of the peaks was modelled using the Caglioti formula and refining the U, V and W parameters. The batch peak fitting was performed using the Gaussian peak shape and the instrumental broadening (calculated using the Caglioti formula for the $LiMn_2O_4$ peak positions) was subtracted from the obtained FWHM values (http://pd.chem.ucl.ac.uk/pdnn/peaks/broad.htm) before performing the first-degree polynomial fitting for the Williamson-Hall plots (FWHM*cos(theta) vs sin(theta)). The results obtained from this spatially-resolved analysis are presented in Supplementary Figure 39. It can be seen that the results are in agreement with the ones obtained from the representative particle (Supplementary Figure 38) as it is shown that the slope (corresponding to strain) increases from XRD-CT dataset 1 to 5 with a small decrease in intercept (corresponding to crystallite size). However, it should be pointed out that one should be careful when interpreting these results as although the Rwp values from the peak fitting are low, the error in the slopes and intercepts is more significant (norm of the residuals) and the number of peaks used can have a strong impact on the obtained values. For these reasons, we maintained our analysis of the cycled cell using the summed diffraction patterns from the three segmented regions rather than performing the spatially-resolved analysis.

The Williamson-Hall plot analysis was also performed for the cycled cell and specifically for the summed diffraction patterns exported from the three segmented regions presented in Figure 7 in the main manuscript. The obtained results are in full agreement with the results obtained from the *in situ* experiment (Supplementary Figure 40). Specifically, it is seen that the offset of the three fitted lines is the same within the experimental errors while the slope is increasing from Segmentation 3 to Segmentation 1. As discussed in the main manuscript, Segmentation 3 corresponds to the region of the sample where there is mainly the $LiMn_2O_4$ cubic spinel present while Segmentation 1 contains the various undesired phases such as the Li_2MnO_3 and the $LiMnO_2$ cubic rock-salt. The obtained values for the three slopes are 0.82, 0.66 and 0.50.

Supplementary References

1. Finegan, D.P. *et al.* Spatially Resolving Lithiation in Silicon–Graphite Composite Electrodes via in Situ High-Energy X-ray Diffraction Computed Tomography. *Nano Lett.* **19**, 3811-3820 (2019).
2. Sommer, C., Straehle, C., Köthe, U. & Hamprecht, F.A. in 2011 IEEE International Symposium on Biomedical Imaging: From Nano to Macro 230-233 (2011).
3. Bianchini, M. *et al.* Spinel materials for Li-ion batteries: new insights obtained by operando neutron and synchrotron X-ray diffraction. *Acta Cryst. Sect. B* **71**, 688-701 (2015).
4. Bianchini, M., Suard, E., Croguennec, L. & Masquelier, C. Li-Rich $Li_{1+x}Mn_{2-x}O_4$ Spinel Electrode Materials: An Operando Neutron Diffraction Study during Li+ Extraction/Insertion. *J. Phys. Chem. C* **118**, 25947-25955 (2014).
5. Choa, J. & Thackeray, M.M. Structural Changes of $LiMn_2O_4$ Spinel Electrodes during Electrochemical Cycling. *J. Electrochem. Soc.* **146**, 3577-3581 (1999).
6. Thackeray, M.M. *et al.* The quest for manganese-rich electrodes for lithium batteries: strategic design and electrochemical behavior. *Sust. Energy & Fuels* **2**, 1375-1397 (2018).
7. Wojdyr, M. Fityk: a general-purpose peak fitting program. *J. App. Cryst.* **43**, 1126-1128 (2010).
